# Calcineurin–NFAT signalling in myeloid leucocytes: new prospects and pitfalls in immunosuppressive therapy

**DOI:** 10.15252/emmm.201707698

**Published:** 2017-06-12

**Authors:** Kamila Bendickova, Federico Tidu, Jan Fric

**Affiliations:** ^1^ Center for Translational Medicine (CTM) International Clinical Research Center (ICRC) St. Anne's University Hospital Brno Brno Czech Republic

**Keywords:** cyclosporine A, Dectin‐1, immunosuppression, tacrolimus, TLR4, Haematology, Immunology

## Abstract

Myeloid leucocytes mediate host protection against infection and critically regulate inflammatory responses in body tissues. Pattern recognition receptor signalling is crucial for myeloid cell responses to pathogens, but growing evidence suggests an equally potent role for Calcineurin–NFAT signalling in control of myeloid cell function. All major subsets of myeloid leucocytes employ Calcineurin–NFAT signalling during immune responses to pathogens and/or tissue damage, but the influence this pathway exerts on pathogen clearance and host susceptibility to infection is not fully understood. Recent data from experimental models indicate that Calcineurin‐NFAT signalling is essential for infection control, and calcineurin inhibitors used in transplantation medicine (including cyclosporine A and tacrolimus) are now being tested for efficacy in a diverse range of inflammatory conditions and autoimmune pathologies. Efforts to repurpose calcineurin inhibitor drugs for new therapeutic applications may yield rapid improvements in clinical outcomes, but the potential impact of these compounds on myeloid cell function in treated patients is largely unknown. Here we discuss Calcineurin–NFAT control of myeloid leucocyte function in the context of recent therapeutic developments and ongoing clinical studies.

**Glossary**
Adaptive immune response: Branch of immune system evolved in vertebrates to provide more specific recognition of dangerous antigens. Lymphocytes adapt to specific pathogen and confer lifelong protective immunity due to immunologic memory of adaptive response. In addition to specificity, the immunologic memory is the major benefit of adaptive immunity as it activates rapid and robust protective response in case of re‐exposure to the same antigen.
Calcineurin inhibitors: Drugs that prevent calcineurin‐driven dephosphorylation and activation of nuclear factor of activated T cells (NFAT). The archetypal drugs in this class are cyclosporine A and tacrolimus which have revolutionized the field of organ transplantation. The main purpose of these inhibitors is to suppress T‐cell responses to allografts.
Drug repurposing: Strategy of testing approved agents for new therapeutic applications. This is an important approach used to accelerate the discovery of new treatment strategies and reduce costs associated with development of novel compounds. Drug repurposing is typically guided by detailed molecular knowledge of the target pathologies.
Fungal morphotypes: Some fungi species including *Aspergillus fumigatus* develop through different morphotypic stages; this includes conidia, swollen conidia and fully germinated hyphae. This process has important consequence for pattern recognition receptor stimulation as *Aspergillus* morphotypes express different amounts of β‐glucans on the surface.
Immune synapse: Cell–cell communication used by immune cells. The formation of immune synapses is the initial event leading to adaptive immunity response and is characterized by membrane rearrangements in both cell types involved.
Immunomodulatory chemokines and cytokines: Signalling compounds secreted by various cells to orchestrate immune response to a desired level, that is immunopotentiation, immunosuppression or induction of immunologic tolerance.
Immunosuppressive therapy: Therapeutic administration of drugs that inhibit immune responses. Immunosuppressive drugs target several different mechanisms and are primarily used to prevent rejection of tissue grafts and transplants as well as suppress autoimmune diseases and inflammatory disorders.
Innate response: Branch of the immune system that allows direct and immediate responses to pathogens. In vertebrates, this evolutionarily conserved host protection strategy is complemented by the adaptive immune system.
Lupus nephritis: Inflammatory disease affecting the kidney and, more specifically, glomeruli. It is caused by systemic lupus erythematosus and can lead to kidney failure. Systemic lupus erythematosus is an autoimmune disease caused by production of autoimmune antibodies against nuclear antigens associated with chronic inflammation.
Myeloid immunity: Immune response mediated by cells of myeloid origin such as granulocytes, macrophages, monocytes and some dendritic cell subsets. Upon pathogen invasion, myeloid cells are rapidly recruited to the site of infection, where they exert their effector functions (cytokine secretion, phagocytosis, etc.).
Ontogeny: All developmental changes occurring throughout the existence of an individual organism. In cell biology, ontogeny refers specifically to developmental and differentiation processes within a cell lineage.
Pattern recognition receptors: Set of innate immune receptors that recognize molecular patterns associated with pathogens or tissue damage. Advances in understanding of their molecular mechanisms of action are leading to the development of new therapies.
Physical form of the antigen: Immunogenicity of soluble and particulate form of antigens is different, for example pattern recognition receptor binds the two forms of β‐glucan differently. Differences in this process following ligation by soluble versus particulate ligands enable receptor‐driven distinction between the two forms of the same antigen.
Plasmacytoid DCs: Subset of dendritic cells specialized in rapid type I interferon production mainly in response to viruses or nucleic acids. pDCs have been implicated in pathogenesis of autoimmune diseases characterized by type I IFN signature.
Rheumatoid arthritis: Chronic autoimmune disorder associated with tissue inflammation of the joints. Several clinical trials have already shown beneficial effects of immunosuppression in patients affected by this condition.
Sjogren's syndrome: Autoimmune disorder characterized by chronic inflammation, infiltration of immune cells to exocrine organs and progressive destruction of moisture‐producing glands.
T‐cell receptor: Complex of integral membrane proteins, which recognizes specific antigen and activate T cells. The variety of TCR is established by developmentally regulated TCR gene rearrangements followed by predominantly intra‐thymic selection processes.

## Introduction

During an adaptive immune response, engagement of the T‐cell receptor stimulates an increase in intracellular calcium that promotes calmodulin binding to the serine/threonine protein phosphatase enzyme calcineurin. Once activated in this way, calcineurin can dephosphorylate nuclear factor of activated T‐cell (NFAT) transcription factors, which modify gene expression and regulate immune responses. The Calcineurin–NFAT pathway was initially described in T cells, where NFAT acts as a master regulator of lymphocyte development, expression of interleukin (IL)‐2, and controls major effector T‐cell functions. Accordingly, drugs developed to inhibit Calcineurin–NFAT binding such as cyclosporine A and tacrolimus have proven highly effective at suppressing T‐cell responses and preventing allograft rejection in transplantation medicine. While long regarded as a specific regulator of T‐cell activity, NFAT signalling was later also identified in other cell types including B cells (Muller & Rao, 2010), and was reported to mediate embryogenic development of multiple tissues including the hematopoietic system (Muller *et al*, 2009). These data suggested that the influence of Calcineurin–NFAT on immunity was not restricted to T cells alone and that potent effects outside the adaptive immune response were likely.

Despite widespread use in clinical settings, the mechanisms by which Calcineurin–NFAT inhibitors suppress host immunity are not as specific as was initially thought. Various studies of cyclosporine A and tacrolimus treatment have revealed a surprising ability of these drugs to disrupt T‐cell activation without impacting on Ca^2+^ flux (Metcalfe *et al*, 1994), and impair lymphocyte responses via effects on the mitogen‐activated protein kinase (MAPK) pathway (Su *et al*, 1994; Matsuda *et al*, 1998) in NFAT‐independent manner. Given that NFAT can also cooperate with transcription factors such as AP‐1 and NF‐kB to modify immune responses (Crabtree, 1989), it is perhaps not surprising that calcineurin inhibitors are now thought to mediate wide‐ranging effects that can also impact on myeloid cell function. The key roles of Calcineurin–NFAT signalling in myeloid cell biology have recently been discussed elsewhere (Muller & Rao, 2010; Fric *et al*, 2012), and myeloid lineages not directly involved in host immunity to pathogens will not be discussed in detail here. The current review instead focuses on Calcineurin–NFAT control of myeloid immunity because the growing number of clinical studies is now seeking to manipulate this pathway for therapeutic benefit in diverse human pathologies. Indeed, all five members of the NFAT family are known to be involved in regulation of immune responses (Macian, 2005), and a major risk of immunosuppressive therapy in human patients is increased susceptibility to opportunistic infections. Consequently, investigators seeking to repurpose calcineurin inhibitor drugs for new disease indications must consider recent reports that NFAT signalling exerts a critical influence on innate immune function, as well as emerging evidence that these therapies disrupt pathways known to be essential for myeloid cell defence against pathogens.

## Pattern recognition receptors initiate Calcineurin–NFAT signalling in myeloid leucocytes

**Figure 1:**
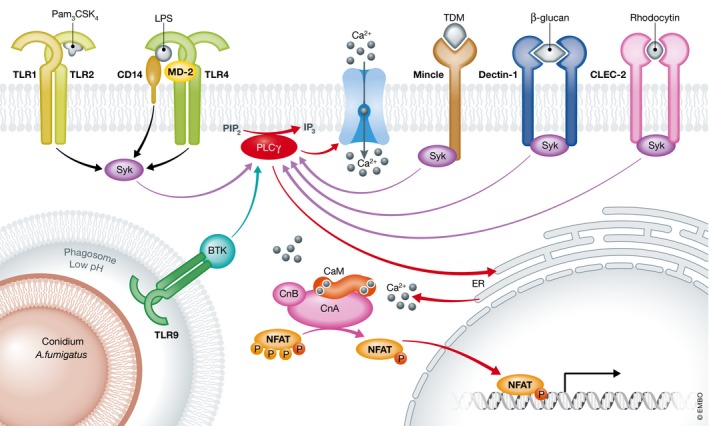
PRR signal integration with the Calcineurin–NFAT pathway Multiple different pattern recognition receptors (PRRs) have been reported to trigger Calcineurin–NFAT signalling upon ligand binding. In particular, TLRs and C‐type lectin receptors (CLR) play critical roles in NFAT activation during innate immune responses. The observation that LPS exposure can stimulate NFAT‐mediated IL‐2 expression focused the majority of early research on the role of TLR4 (Granucci *et al*, 2001, 2003). The LPS co‐receptor CD14 was later also reported to promote Calcineurin–NFAT activation via recruitment of Syk (Zanoni *et al*, 2009). In mast cells, heterodimers of TLR1‐TLR2 recognize Pam_3_ CSK_4_ and drive the recruitment of Fc‐γ receptor which contains ITAM motifs (Jin *et al*, 2016). TLR9 can also promote NFAT activation upon exposure to *Aspergillus fumigatus* conidia in acidified endosomes, which results in recruitment of BTK, activation of PLCγ followed by increase in intracellular calcium concentration and NFAT translocation to the nucleus (Herbst *et al*, 2015). Alternatively, Syk recruitment, PLCγ activation and nuclear localization of NFAT can instead be induced by ligand binding of ITAM‐containing CLRs such as Dectin‐1 (Goodridge *et al*, 2007), macrophage‐inducible Ca^2+^‐dependent lectin (Mincle) (Yamasaki *et al*, 2008) and CLEC‐2 (Mourao‐Sa *et al*, 2011; Severin *et al*, 2011).

NFAT activation in dendritic cells (DCs) and macrophages was first observed upon cell activation through Dectin‐1 (Goodridge *et al*, 2007), and was soon followed by reports that NFAT activation could also be triggered by other pattern recognition receptors (PRRs) including TLR4 and CD14 (Fig 1).

Initial mechanistic studies sought to understand how NFAT regulates gene expression in DCs exposed to various types of pathogen (Granucci *et al*, 2001, 2003). In particular, much early research focused on the role of the CD14‐MD2‐TLR4 pathway in modulating NFAT signalling (Zanoni *et al*, 2009). This critical signalling axis is now known to exert complex effects on DC function via a range of different mechanisms, including LPS binding to CD14, which can induce calcium flux without the involvement of TLR4 (Zanoni *et al*, 2009), and internalization of the entire CD14‐MD2‐TLR4 complex which instead promotes interferon production (Kagan *et al*, 2008; Zanoni *et al*, 2011). Subsequent work has suggested that a large number of innate cell functions could be controlled by protein complexes such as these termed “signalling organelles” which cooperate to alter intracellular calcium levels and regulate gene expression (Zanoni *et al*, 2011; Chiang *et al*, 2012; Kagan, 2012). While it is now widely accepted that calcineurin can dephosphorylate NFAT to facilitate transcription factor translocation to the nucleus in myeloid cells as well as in lymphocytes, two important questions remain unresolved (i) which PRRs are involved in mediating this process? and (ii) why are NFAT‐driven expression programs highly specific to different cells and tissues?

Calcineurin–NFAT activity has now been reported in almost all subsets of myeloid cells (Muller & Rao, 2010; Fric *et al*, 2012). Most recently, an important role of NFAT1 was identified in microglia subjected to chronic LPS stimulation (Ma *et al*, 2015). Other investigators have dissected the translocation/turnover kinetics of the dephosphorylated nuclear fraction of NFAT3 and 4 in monocytes activated through TLR2/4 (Minematsu *et al*, 2011). In human mast cells, calcium mobilization is activated by triggering of TLR‐2, which has been linked with NFAT‐mediated transcriptional responses to *Leishmania* (Zaidi *et al*, 2006; Bhattacharjee *et al*, 2016). Calcineurin–NFAT signalling has also been shown to regulate mast cell activation (Walczak‐Drzewiecka *et al*, 2008), survival (Ulleras *et al*, 2008) and cytokine expression (Monticelli *et al*, 2004; Klein *et al*, 2006). Furthermore, recognition of bacterial or fungal entry into host cells via cytoplasmic PRRs known as NOD‐like receptors (NLRs) has also been shown to influence Calcineurin–NFAT activation (Tourneur *et al*, 2013; Vandewalle *et al*, 2014). The various PRR‐dependent mechanisms of Calcineurin–NFAT activation identified in myeloid leucocytes are summarized in Fig 1.

## Features of the Calcineurin–NFAT signalling cascade in myeloid cells

A key advance in understanding how PRR ligation triggers Calcineurin–NFAT signalling was the discovery that cytoplasmic calcium levels are the sole determinant of calcineurin activity following leucocyte exposure to LPS (Zanoni *et al*, 2009) or the Dectin‐1 ligand curdlan (Xu *et al*, 2009). While originally described in signalling downstream of the T‐cell receptor, immune receptor tyrosine‐based activation motifs (ITAM) are now also known to contribute to signal transduction in myeloid cells upon ligation of Dectin‐1 and other C‐type lectin receptors including CLEC‐2 (Mourao‐Sa *et al*, 2011; Severin *et al*, 2011).

In a signalling cascade comparable with that displayed by T cells, myeloid cells undergo dephosphorylation of ITAM motifs by Src kinases to create a binding site for spleen tyrosine kinase (Syk), which is the major integrator of PRR signals that induce intracellular calcium flux. These shared early signalling events suggest that Dectin‐1 initiation of phagocytic activity in myeloid cells is the functional equivalent of immune synapse formation for T‐cell stimulation (Goodridge *et al*, 2011). Syk subsequently cooperates with Src kinases to activate phospholipase γ (PLCγ), which represents the major point of convergence between PRR signalling and Calcineurin–NFAT activation by stimuli including Dectin‐1 ligands (Xu *et al*, 2009). Evidence that PLCγ plays a major role in DC biology was first reported by Tassi *et al*, who observed that bone marrow‐derived DCs (BMDCs) derived from PLCγ‐deficient mice were unable to prime T‐cell expression of IL‐17 in response to Dectin‐1 binding (Tassi *et al*, 2009). PLCγ also contributes to myeloid cell ontogeny due to activation by the key growth factors M‐CSF and G‐CSF in lineage progenitor cells (Barbosa *et al*, 2014).

An important signalling event in NFAT activation is ligand internalization and/or intense clustering of signalling molecules to lipid rafts. This process can be readily observed in response to molecules in particulate form such as β‐glucan or zymosan (Goodridge *et al*, 2007), but has also been shown to occur following a LPS challenge. CD14‐mediated internalization of LPS into signalling compartments activates Syk/PLCγ and promotes intracellular calcium flux in monocytes, macrophages and DCs (Zanoni *et al*, 2011; Vigano *et al*, 2015). We and others have also reported that signalling through PRRs can induce calcium flux and activate Calcineurin–NFAT signalling upon recognition of complex particulate antigens including whole bacteria and fungal conidia (Fric *et al*, 2014b). Indeed, while zymosan binding to Dectin‐1 alone is sufficient to activate Calcineurin–NFAT signalling, *Aspergilus fumigatus* can also trigger TLR9 and Bruton's tyrosine kinase (Btk) in addition to Dectin‐1, leading to further upregulation of Calcineurin–NFAT activity (Strijbis *et al*, 2013; Herbst *et al*, 2015).

## Calcineurin–NFAT control of bacterial and viral infections

Engagement of the Calcineurin–NFAT pathway has now been detected in myeloid cell responses to bacteria (Zanoni *et al*, 2009; Minematsu *et al*, 2011; Ranjan *et al*, 2014), parasitic infections (Kayama *et al*, 2009) and viruses (Miskin *et al*, 1998, 2000). In human DC, exposure to cyclosporine A reduces interferon (IFN)‐α responses to Sendai virus (Tajima *et al*, 2003), while macrophages from NFAT4 knockout mice display reduced iNOS expression and attenuated bactericidal activity in a sepsis model, consistent with the ability of calcineurin inhibitors to block NFAT4 binding to the iNOS promoter (Ranjan *et al*, 2014). Given that the NFAT family can also cooperate with transcription factors such as NF‐κB to modulate gene expression (Bronk *et al*, 2014), the Calcineurin–NFAT pathway is also likely to impact on antimicrobial immune responses via additional mechanisms that have yet to be identified. Indeed, previous studies of NFAT‐dependent genes have relied mainly on global gene expression analyses in genetically engineered mouse models; hence, there are currently only limited data available on NFAT binding sites in myeloid cells. However, using an alternative chip‐on‐chip approach, Yu *et al* performed genome‐wide mapping of potential target sites in human DCs to identify that NFAT1 can modulate expression of critical immunomodulatory chemokines and cytokines including IL‐2, IL‐12p40 and IL‐23 (Yu *et al*, 2015). Similar strategies have also been used to identify cooperation between NFAT4 and transcription factor IRF7 in binding to IFN promoters in plasmacytoid DCs (Bao *et al*, 2016).

So far there are only limited data available from studies of human tissues and patient samples to support a role for NFAT in myeloid cell responses to major pathogens *in vivo*. However, signalling through TLR4 and NOD1, which impact on the Calcineurin–NFAT pathway, correlate with phagocytic activity in human renal transplant recipients who exhibit increased susceptibility to *E. coli* infection (Tourneur *et al*, 2013). When combined with extensive data available from experimental models, these findings provide clear evidence that Calcineurin–NFAT signalling in myeloid cells is critically involved in host protection against infection. It is therefore highly likely that drug inhibition of Calcineurin–NFAT signalling in myeloid cells will disrupt numerous mechanisms of innate immune protection in treated human patients.

## Calcineurin–NFAT‐regulated genes in myeloid cells control fungal infection

The first NFAT‐regulated genes to be identified included IL‐2 expression induced by triggering of the T‐cell receptor (Shaw *et al*, 1988). An important observation in this context was that stimulation of DCs with microbial compounds can also induce NFAT‐dependent production of IL‐2 (Granucci *et al*, 2001, 2003; Zanoni *et al*, 2009). While both NFAT‐ and MAPK‐dependent effects of calcineurin inhibition on myeloid cell development were also identified, these were not initially linked with altered host susceptibility to infection (Miranda & Johnson, 2007; Fric *et al*, 2014a). Indeed, Calcineurin–NFAT‐driven IL‐2 expression can also be triggered by DC phagocytosis of sterile adjuvants including alum, SiO_2_ particles and monosodium urate crystals (Khameneh *et al*, 2017).

A role for calcium‐NFAT signalling in innate immunity was initially identified in NK cells (Aramburu *et al*, 1995), and later reported in macrophages and DCs stimulated through Dectin‐1 using either *Candida albicans* or zymosan, which modulated expression of key transcription factors (Egr2, Egr3) and pro‐inflammatory mediators including Cox‐2, IL‐2, IL‐10 and IL‐12p70 (Goodridge *et al*, 2007; Xu *et al*, 2009). These data suggest that a wide range of effects can be mediated by calcium‐NFAT signalling, especially via DC‐derived IL‐2 which has been identified as a key mediator of NK cell activation (Granucci *et al*, 2004, 2006) and regulatory T‐cell (T‐reg) maintenance (Guiducci *et al*, 2005). Indeed, recent work using an intranasal *Aspergillus fumigatus* infection model has demonstrated an important role for DC‐derived IL‐2 in mucosal immune responses *in vivo* (Zelante *et al*, 2015). In this report, conditional knockout of IL‐2 expression in CD11c^+^ cells was sufficient to disrupt fungus recognition, inhibit phagocytosis and impair Th17 responses to live conidia. Correlation of NFAT signalling with DC production of the antifungal protein pentraxin‐3 has been also reported in a model of intravenous *A. fumigatus* infection (Zelante *et al*, 2017). Consequently, host deficiency in calcineurin signalling or lack of IL‐2 expression in myeloid cells confers a significant increase in *A. fumigatus*‐associated mortality in rodents (Zelante *et al*, 2015).

While IL‐2 cytokine clearly has a major role to play in regulating cross talk between innate and adaptive immune cells (Malek & Castro, 2010; Yuan *et al*, 2015), other NFAT‐regulated genes involved in host protection against infection include Cox‐2, PGE‐2 and various immunomodulatory cytokines (Zanoni *et al*, 2012). For example, tacrolimus treatment leads to a significant decrease in TNF expression by murine macrophages infected with *A. fumigatus* (Herbst *et al*, 2015). These data demonstrate that Calcineurin–NFAT signalling not only represents a central component of adaptive immunity but also plays important roles in innate responses. Consequently, the increased risk of bacterial and fungal infections observed in transplant patients treated with calcineurin inhibitors is likely due to defects in innate pathogen recognition in addition to suppression of T‐cell responses.

## Myeloid Calcineurin–NFAT function in immunopathology

Myeloid cells accumulate in host tissues and organs in a range of different pathologies including chronic inflammatory disorders, malignancies and autoimmune diseases (Nicholson *et al*, 2009). PRR signalling in myeloid cells has been the target of rheumatoid arthritis (RA) experimental therapy (Kim *et al*, 2014). Escolano *et al* (2014) showed that calcineurin impairment in macrophages drives the development of an anti‐inflammatory phenotype, with potential beneficial effects in some pathologies. Critical to many of these disease processes is antigen presentation by monocytes, macrophages and DCs, which stimulate T cells to produce pro‐inflammatory cytokines and mediate potent effector functions. While myeloid cell induction of T‐cell‐mediated immunity is critical for eliminating pathogens from infected tissues, these responses can also damage host tissues if directed against self‐antigens or not subsequently resolved. The range of clinical settings in which calcineurin inhibitor drugs might prove efficacious is therefore extremely broad, and the potential side effects likely include elevated risk of a wide variety of infections (Table 1).

**Table 1:** Overview of experimental models demonstrating a role for NFAT in innate immunity

	Model Disease	Cell type	Host	Pathogen Trigger	Study	Inhibitor Gene KO	Genes, Phenotype	References
FUNGI	Invasive fungal infection	BMDM BMDC	Ms	*C. albicans*	*In vitro*	CsA VIVIT	Egr2, Egr3, Cox‐2, IL‐2, IL‐10, IL‐12p70	Goodridge *et al* (2007)
		Neut MF	Ms	*C. albicans*	*In vivo In vitro*	CsA CnB NFAT2 NFAT4	Mortality	Greenblatt *et al* (2010)
		Al. MF	Ms	*A. fumigatus*	*In vivo*	FK506	Hyperinflammation Mortality	Herbst *et al* (2013)
		DC	Ms	*A. fumigatus*	*In vivo In vitro*	CsA FK506 CnB	Mortality, IL‐2, Th17 regulation	Zelante *et al* (2015)
		Neut MF	Ms Hu Zf	*A. fumigatus*	*In vivo In vitro*	FK506	Mortality	Herbst *et al* (2015)
		DC Neut MF	Ms	*A. fumigatus*	*In vivo In vitro*	CnB	Mortality, Ptx‐3	Zelante *et al* (2017)
		Neut	Hu	*A. fumigatus*	*Ex vivo In vitro*	CsA FK506	*A.f*. growth control	Imbert *et al* (2016)
		MF	Hu Zf	*A. fumigatus*	*In vivo In vitro*	FK506	Inflammatory response A.f. growth control	Shah *et al* (2016)
YEAST	Opportunistic infection	MF	Ms	*S. cerevisiae*	*In vivo, In vitro*	CsA FK506	CCR2 chemokines, clearance of infection	Busch *et al* (2016)
BACTERIA/PAMPs	Sepsis	MF	Ms	LPS	*In vivo In vitro*	CsA, VIVIT	iNOS Bactericidal activity	Ranjan *et al* (2014)
	Colitis IBD	MF	Ms	LPS	*In vivo In vitro*	FK506 VIVIT	IL‐12p40 IL‐12p70 IL‐23	Elloumi *et al* (2012)
	Acute pyelonephritis	Neut DC MF	Ms Hu	*E. coli*	*In vivo In vitro*	CsA VIVIT	Chemokines Susceptibility	Tourneur *et al* (2013)
	Bacterial infection	Mo MF DC	Ms	LPS	*In vivo In vitro*	FK506 CnB	LPS tolerance	Jennings *et al* (2009) Kang *et al* (2007)
PARASITES	Chagas' disease	BMDC RAW 264	Ms	*T. cruzi*	*In vivo In vitro*	FK506 NFAT2	TLR‐independent, calcium‐dependent, IFN‐g production	Kayama *et al* (2009)
VIRUSES	African swine fever	MF	Pig	*African swine fever virus*	*In vitro*	CnA deletion	Virus protein inhibits CnB activity	Miskin *et al* (2000) Miskin *et al* (1998)
	Sendai virus	DC	Hu	*Sendai virus*	*In vitro*	CsA	IFN‐α	Tajima *et al* (2003)
STERILE PARTICLES	Vaccination	DC	Ms	Alum adjuvants	*In vivo In vitro*	CsA FK506	IL‐2, CD4 proliferation, Humoral response	Khameneh *et al* (2017)

Species abbreviations: mouse (Ms), human (Hu), *Candida* (C.), *Aspergillus fumigatus* (A.f.), trypanosoma (T.). Cell types: bone marrow‐derived macrophages (BMDMs), bone marrow‐derived dendritic cells (BMDCs), neutrophils (Neut), alveolar macrophages (Al. MF), monocytes (Mo), cyclosporine A (CsA), tacrolimus (FK506), calcineurin (Cn).

Invasive fungal infections are a major cause of morbidity and mortality in immunocompromised patients (Pappas *et al*, 2010), and both experimental and clinical studies have demonstrated that the Calcineurin–NFAT pathway collaborates with NF‐κB to coordinate macrophage TNF responses to *Aspergillus* (Herbst *et al*, 2015). Similarly, administration of calcineurin inhibitors is associated with defects in neutrophil/macrophage control of fungal germination and hyphal growth that likely increase pathology in transplant recipients (Imbert *et al*, 2016; Shah *et al*, 2016). The discovery that defective Calcineurin–NFAT signalling in myeloid cells confers increased susceptibility to *C. albicans* infection in mice (Greenblatt *et al*, 2010) has also led to the development of new models of infection in immunocompromised hosts that more closely resemble human clinical scenarios. In one such model of pulmonary aspergillosis, disruption of Calcineurin–NFAT signalling impaired fungal killing in alveolar macrophages, leading to sustained inflammatory responses in the lung, enhanced tissue destruction and increased rates of mortality (Herbst *et al*, 2013, 2015). An alternative model of invasive aspergillosis has also been used to demonstrate that NFAT‐dependent expression of IL‐2 in DCs is required for optimal Th17 responses in the lung, which decreases inflammatory pathology and reduces risk of mortality following infection (Zelante *et al*, 2015, 2017). In the peritoneal cavity, calcineurin inhibition during opportunistic *Saccharomyces cerevisiae* infection was reported to impair macrophage expression of CCR2 chemokines, impair neutrophil mobilization and delay pathogen clearance (Busch *et al*, 2016). The process of phagocytosis itself has also been identified as a driver of NFAT activation in macrophages, which may employ the TLR9‐BTK‐Calcineurin–NFAT (MyD88‐independent) pathway as a mechanism of intracellular pathogen detection (Herbst *et al*, 2015). There is now substantial evidence that Calcineurin–NFAT signalling in myeloid cells affects the outcome of fungal infections in particular, although only limited data are available on how myeloid cell responses to fungi are altered in human patients receiving immunosuppressive therapy. Indeed, drug effects on non‐immune lineages including epithelial cells that also express fungus‐sensing PRRs may further modify patient outcomes. Indeed, a recent study demonstrated that upregulation of Dectin‐1 in airway epithelial cells enhanced pro‐inflammatory responses to *A. fumigatus*, increased neutrophil recruitment to the lungs and improved rates of *Aspergillus* clearance and host survival (Liu *et al*, 2015). However, it remains unconfirmed whether this Dectin‐1‐dependent ability of epithelial cells to protect against fungal infection is mediated by NFAT.

## Autoimmune disorders as targets for immunosuppressive therapies

The discovery that drug inhibition of the Calcineurin–NFAT pathway potently suppresses T‐cell responses led to a revolution in transplant medicine (Yeh & Markmann, 2013). While clinical usage of calcineurin inhibitors began in the 1980s, additional effects of these drugs on myeloid cell biology were not appreciated until almost 20 years later. Extensive efforts are now being made to better understand the full range of effects induced by calcineurin inhibitors and to identify new therapeutic applications for these drugs in a wide range of disease indications. While there is huge potential to improve therapy options for several major human disorders, these strategies will likely also confer increased infection risk due to disruption of myeloid cell responses to commensal organisms and pathogenic microbes.

**Figure 2:**
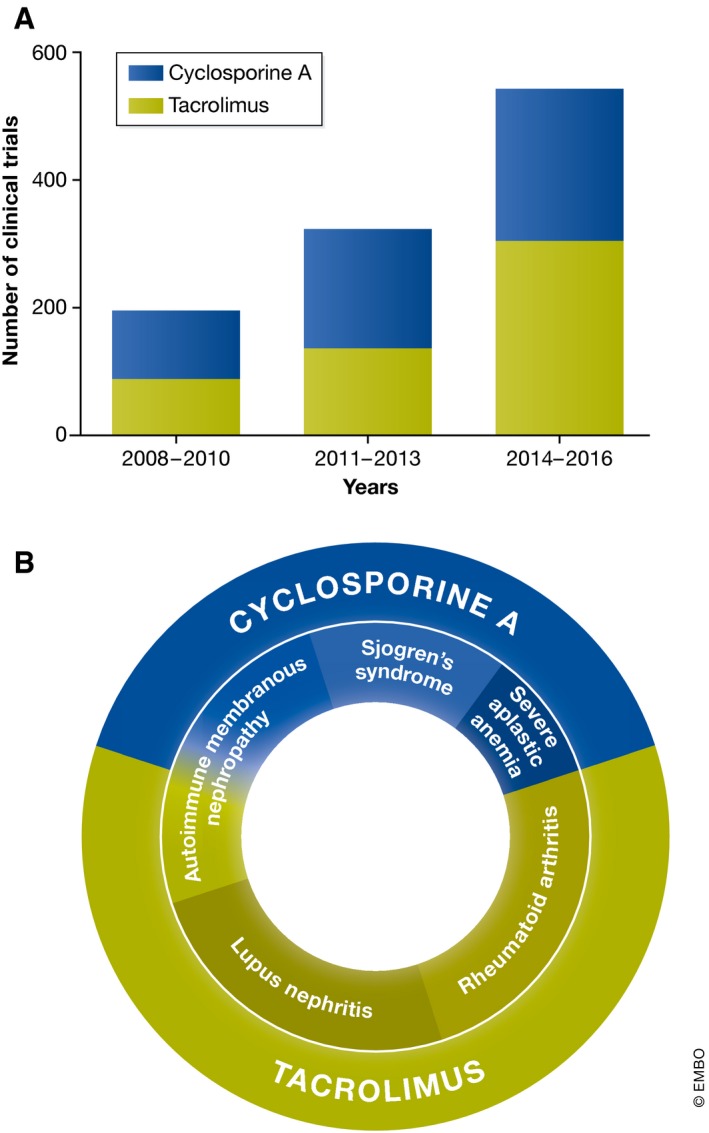
Clinical testing of calcineurin inhibitors cyclosporine A and tacrolimus in autoimmune disorders (A) Increasing number of clinical studies testing “off‐label” calcineurin inhibitor use for the treatment of autoimmune disorders. Histograms show studies of tacrolimus and cyclosporine A as well as the year each study was completed or last updated (clinicaltrials.gov). (B) Graphical overview of the major autoimmune disorders targeted using calcineurin inhibitors.

In a previous study of calcineurin inhibitor effects on macrophage function in murine colitis, LPS‐induced expression of IL‐12p40, IL‐12p70 and IL‐23 was significantly reduced by treatment with tacrolimus or the calcineurin inhibitory peptide 11R‐VIVIT (Elloumi *et al*, 2012). These data suggest that selective blockade of NFAT might be a promising therapeutic strategy in inflammatory bowel diseases. These findings are consistent with an earlier report that inactivation of calcineurin in myeloid cells can induce a form of LPS tolerance that inhibits inflammation (Jennings *et al*, 2009). Administration of calcineurin inhibitors may therefore also provide effective protection against LPS toxicity in disorders such as sepsis. Indeed, the range of pathologies in which calcineurin inhibition is thought to be potentially beneficial is now extensive, and the number of clinical studies initiated to test the efficacy of cyclosporine or tacrolimus has seen a corresponding increase in recent years (Fig 2A).

A potential new therapeutic application for calcineurin inhibitor drugs is the treatment of autoimmune disorders (Fig 2B and Table 2). Several studies have now reported that cyclosporine A can inhibit pathological IL‐17 expression in patients with RA (Zhang *et al*, 2008). While some studies have failed to show any improvement of advanced RA with tacrolimus treatment (Schiff *et al*, 2010), recent clinical studies using tacrolimus as part of a multiple drug treatment in RA have reported very promising results (Takahashi *et al*, 2015; Hirai *et al*, 2017; Naniwa *et al*, 2017). However, since clinical studies often exclude patients who develop infections of unconfirmed origin, it will be important to determine whether treated individuals exhibit increased infection risk as reported elsewhere in immunosuppressed arthritis patients (Misra *et al*, 2014).

**Table 2:** Overview of clinical studies using calcineurin inhibitors to treat autoimmune disorders

Pathology	Inhibitor	Monotherapy	In combination
Rheumatoid arthritis	FK506	NCT02837978 NCT01511003	Methotrexate—NCT02837978 Methotrexate—NCT01746680 Sarilumab—NCT02373202 Biolagens—NCT01870908
Membranous nephropathy	FK506		Rituximab—NCT00843856 Mycophenolate—NCT01955187
	CsA	NCT01282073 NCT01180036	Rituximab NCT00977977
Lupus nephritis	FK506	NCT01410747 NCT01316133 NCT02457221 NCT01580865 NCT02630628	
Severe aplastic anaemia	CsA		Eltrombopag + hATG NCT01623167 hATG or rAGT NCT00260689 Alemtuzumab—NCT00195624
Sjogren's syndrome	CsA	NCT02004067 NCT02370550	

Both cyclosporine A and tacrolimus have proven effective for treatment of autoimmune membranous nephropathy, which is increasingly prevalent in older patients who already exhibit high risk of opportunistic infections (Yamaguchi *et al*, 2014; Alfaadhel & Cattran, 2015). For other autoimmune pathologies including lupus nephritis, clinical studies are ongoing to demonstrate the efficacy of tacrolimus treatment which is currently used only on an “off‐label” basis (Kraaij *et al*, 2016). Similarly, the chronic autoimmune condition known as Sjogren's syndrome is reportedly associated with abnormal activation of Th17 cells and has also been shown to respond well to combination therapy including cyclosporine A. Together, these data demonstrate that cyclosporine and tacrolimus can provide therapeutic benefit in human rheumatoid disorders and potentially other diseases in which myeloid cell function has been implicated.

Numerous *in vitro* studies and investigations in animal models have indicated that calcineurin inhibition can potently inhibit inflammatory cytokine expression without inducing severe side effects. While the range of potential drug effects on host susceptibility to infection appears extensive, additional effects of calcineurin inhibitors on myeloid cell activity in autoimmune disease are also likely to be identified in future. Indeed, the potent immunosuppressive properties of myeloid cells have already been confirmed in studies of tolerogenic DCs in RA (Hilkens *et al*, 2010; Hilkens & Isaacs, 2013) and via development of novel tissue‐specific DC‐based immunotherapies (Mbongue *et al*, 2014). Further investigations will therefore be required to better understand the role of Calcineurin–NFAT signalling in control of myeloid cell function in human patients, to target this pathway more effectively in future and achieve greater therapeutic benefit for patients with a range of increasingly common pathologies.

## Concluding remarks

In the current review, we have summarized recent advances in understanding the role played by calcineurin/NFAT‐regulated genes in the control of myeloid leucocyte responses to infection. Due to decades of clinical use in transplant settings, there is little concern regarding the safety of calcineurin inhibitor drug use in human patients. While most of the clinical studies discussed in this review carefully assessed infection risk associated with immunosuppressive therapies, the contribution of myeloid cell dysfunction in treated patients has been largely overlooked. To our knowledge, no previous study has reported a link between immunosuppressive therapy and dysregulation of myeloid immunity. However, data from infection models and studies in genetically engineered animals clearly indicate a major role for Calcineurin–NFAT signalling in myeloid cell protection against pathogens.

**Pending issues**

- How far are drug effects on myeloid cells responsible for the increased infection risk observed in patients treated with calcineurin inhibitors?
- How can new knowledge of the roles played by Calcineurin–NFAT signalling be effectively translated into novel therapeutic strategies?
- How do calcineurin inhibitors impact on development and maintenance of different myeloid cells populations in immunosuppressed patients?

## Conflict of interest

The authors declare that they have no conflict of interest.
